# Electromagnetic Wave Shielding Properties of Amorphous Metallic Fiber-Reinforced High-Strength Concrete Using Waveguides

**DOI:** 10.3390/ma14227052

**Published:** 2021-11-20

**Authors:** Sangkyu Lee, Gyuyong Kim, Hongseop Kim, Minjae Son, Yaechan Lee, Yoonseon Choi, Jongmyung Woo, Jeongsoo Nam

**Affiliations:** 1 Department of Architectural Engineering, Chungnam National University, 99 Daehak-ro, Yuseong-gu, Daejeon 34134, Korea; lsg2357@naver.com (S.L.); minjae931226@naver.com (M.S.); cks6832@naver.com (Y.L.); j.nam@cnu.ac.kr (J.N.); 2 Building Safety Research Center, Department of Living and Built Environment Research, Korea Institute of Civil Engineering and Building Technology, 283, Goyang-daero, Ilsanseo-gu, Goyang-si 10223, Korea; hongseopkim@kict.re.kr; 3 Department of Radio and Information Communications Engineering, Chungnam National University, 99 Daehak-ro, Yuseong-gu, Daejeon 34134, Korea; yschoi0703@o.cnu.ac.kr (Y.C.); jmwoo@cnu.ac.kr (J.W.)

**Keywords:** electromagnetic wave, amorphous metallic fiber, shielding effectiveness, waveguide

## Abstract

In this study, high-strength concrete containing hooked-end steel or amorphous metallic fibers was fabricated, and the electrical conductivity and electromagnetic shielding effectiveness were evaluated after 28 and 208 days based on considerations of the influences of the moisture content. Amorphous metallic fibers, which have the same length and length/equivalent diameter ratio as hooked-end steel fibers, were favored for the formation of a conductive network because they can be added in large quantities owing to their low densities. These fibers have a large specific surface area as thin plates. The electromagnetic shielding effectiveness clearly improved as the electrical conductivity increased, and it can be expected that the shielding effectiveness will approach the saturation level when the fiber volume fraction of amorphous metallic fibers exceeds 0.5 vol.%. Meanwhile, it is necessary to reduce the amount of moisture to conservatively evaluate the electromagnetic shielding performance. In particular, when 0.5 vol.% of amorphous metallic fibers was added, a shielding effectiveness of >80 dB (based on a thickness of 300 mm) was achieved at a low moisture content after 208 days. Similar to the electrical conductivity, excellent shielding effectiveness can be expected from amorphous metallic fibers at low contents compared to that provided by hooked-end steel fibers.

## 1. Introduction

The rapid development of information and communication technology has increased the use of electronic devices, thereby causing electromagnetic wave pollution [1,2,3]. In recent years, smart city infrastructure has been constructed worldwide based on various information and communication technologies. One of the key elements required for the operation of a smart city is platform operation via the integrated use and management of data. This implies that the scale of risks in smart cities may differ from that in the existing cities if electromagnetic interference disrupts the operations of various electronic and communication systems. 

Electromagnetic waves may cause the malfunction of electronic devices and can be used for network hacking or as military weapons (e.g., electromagnetic pulses (EMP)) to neutralize major networks of the enemy [4,5,6,7]. It was also reported that long-term exposure to electromagnetic waves can have a harmful impact on human health [8,9,10]. In this regard, research on construction materials with shielding performance is required.

Cement-based composites, which are commonly used construction materials, have some conductivity owing to ionic conduction [11], but it is difficult to expect sufficient shielding performance because their electrical conductivity is low. Therefore, the shielding effectiveness can be improved by increasing the electrical conductivity. This can be achieved by adding conductive materials that decrease the intensities of electromagnetic waves [3,11,12]. Accordingly, studies have been conducted to secure the electrical conductivity and electromagnetic shielding effectiveness by adding various conductive materials, such as metal fibers, carbon fibers, and carbon nanotubes (CNTs), into concrete.

Many studies have been conducted on carbon-based materials, such as CNTs and graphene, as mixing materials for electromagnetic wave shielding owing to their large specific surface area, low density, and excellent mechanical and electrical properties, but they have shortcomings, such as high production cost and low dispersibility [13,14,15]. In the case of metal materials, highly conductive silver, copper, nickel powder, and steel slag were used in the early days of research, but had weaknesses, such as high price, corrosion, increased load, and decreased mechanical strength [2,11,16,17]. To address these problems, studies have been conducted to secure a conductive network using metal fibers with a high aspect ratio [11,18,19,20]. In the case of typical steel fibers, however, their heavy weight still increases the self-weight of the structure, but their actual application is difficult owing to the corrosion problem [21,22]. Their small specific surface area and high density also make it difficult to construct continuous conduction paths, and the complex manufacturing processes, such as casting and hot and cold rolling, are not favorable in terms of carbon dioxide emissions [23].

As described, carbon materials, such as CNTs and graphene, have been researched as major mixing materials for providing electromagnetic performance; despite their shortcomings, they exhibit excellent physical properties compared to the existing metal materials. However, only a few studies have been conducted on metal materials, such as steel fibers. Therefore, this study has focused on amorphous metallic fibers that can complement the shortcomings of typical steel fibers.

Given that amorphous metallic fibers have a lower density than typical steel fibers, they can be secured in larger quantities at the same content and have excellent corrosion resistance [24,25,26]. They can also be favorable for forming a conductive network because they have a large specific surface area as thin plates with a rough surface. In addition, amorphous metallic fibers can reduce carbon dioxide emissions by around 20% as the process used to produce them is simplified compared with typical steel fibers [23]. Previous studies related to amorphous metallic fiber-reinforced concrete, however, were mostly focused on mechanical properties and durability [23,24,25,26,27,28,29,30]. Furthermore, there are few studies related to special physical impacts, such as electromagnetic properties.

Therefore, in this study, hooked-end steel fibers that have been extensively used as construction materials were set as the comparison group so that the electromagnetic shielding effectiveness of amorphous metallic fibers could be compared and evaluated. High-strength concrete containing hooked-end steel or amorphous metallic fibers was then fabricated, and the electrical conductivity and electromagnetic shielding effectiveness were evaluated after 28 and 208 days based on considerations of the influences of moisture content. The electrical conductivity was measured using a digital multimeter, and the electromagnetic shielding effectiveness was measured using waveguides. The effects of the content and specific surface area of fibers on the electrical conductivity and electromagnetic shielding effectiveness were examined, and the relationship between the electrical conductivity and electromagnetic shielding effectiveness was analyzed within the scope of this study. 

## 2. Experimental Design and Method

### 2.1. Materials 

**Figure 1:**
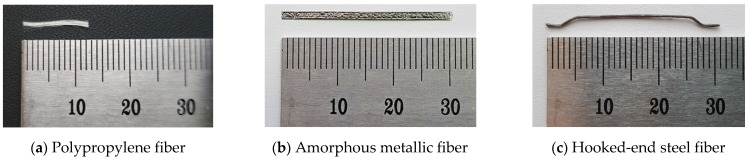
Types of used fibers.

Table 1 and Figure 1 show the physical properties and geometry of the fibers used. Polypropylene fibers are cylindrical with a length of 15 mm, diameter of 20 µm, density of 0.91 g/cm^3^, and a melting point of 170 °C. Amorphous metallic fibers have a thin plate shape with a rough surface and they have a length of 30 mm, width of 1.6 mm, thickness of 29 µm, density of 7.2 g/cm^3^, tensile strength of 1400 MPa, and a specific surface area of 9.6 m^2^/kg. Hooked-end steel fibers are cylindrical with both ends bent in the shape of a hook. They have a length of 30 mm, diameter of 0.25 mm, density of 7.85 g/cm^3^, tensile strength of 1140 MPa, and a specific surface area of 1.0 m^2^/kg. 

Table 2 shows the physical properties of the materials used. Cement (ordinary Portland cement, density: 3150 kg/m^3^, fineness: 320 m^2^/kg), silica fume (density: 2500 kg/m^3^, fineness: 20,000 m^2^/kg), and ground granulated blast-furnace slag (density: 2500 kg/m^3^, fineness: 600 m^2^/kg) were used as a binder. Crushed granitic aggregate (maximum size: 20 mm, density: 2700 kg/m^3^, absorption: 0.9%) was used as coarse aggregate, and river sand (density: 2650 kg/m^3^, absorption: 1%, fineness modulus: 2.6) as fine aggregate. Polycarboxylic acid-type superplasticizer was used as a water-reducing agent.

### 2.2. Experimental Plan and Mixture Proportions

Table 3 shows the experimental plan. In previous studies, fire-resistance performance was evaluated to utilize amorphous metallic fiber-reinforced concrete as a multifunctional material, and this property was linked with the specimen levels tested in this study [27,28]. The specimens had six levels, and 0.15 vol.% of polypropylene fibers was added. Specifically, 0.3 and 0.5 vol.% were added for hooked-end steel fibers and 0.1, 0.3, and 0.5 vol.% for amorphous metallic fibers. The compressive strength, flexural strength, electrical conductivity, and electromagnetic shielding effectiveness were evaluated, and the results were compared and analyzed according to the fiber type and content. 

Table 4 shows the mixed proportions of fiber-reinforced high-strength concrete. Water/Binder (W/B) was set to 0.19 and S/a to 0.45 to fabricate high-strength concrete with a compressive strength of approximately 100 MPa. All specimens satisfied the target slump flow (650 ± 50 mm) and target air content (2 ± 1%).

### 2.3. Preparation of Specimens

Fiber-reinforced high-strength concrete was prepared by adding water and the superplasticizer after the dry mixing of the aggregates and binders. Fibers were then added and sufficiently mixed, and concrete was poured into specimen molds. The prepared specimens were first cured in a water tank (20 ± 2°C) for 28 days and then under constant temperature and humidity conditions (temperature: 20 ± 2 °C, relative humidity: 60 ± 5%) for 180 days. The moisture content of the specimens was calculated using Equation (1) based on the report of RILEM committee TC 129 [31]. The results are listed in Table 5.

(1) $$W_{moisture}=\frac{W_{before\;dry}-W_{after\;dry}}{W_{before\;dry}}\times 100$$

where $W_{moisture}$ is the moisture content (%), $W_{before\;dry}$ is the weight of the specimen before drying (g), and $W_{after\;dry}$ is the weight of the dried specimen (g). 

**Figure 2:**
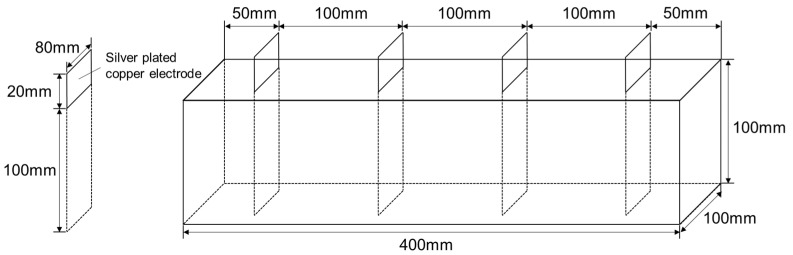
Specimen geometry used for resistance measurements.

**Figure 3:**
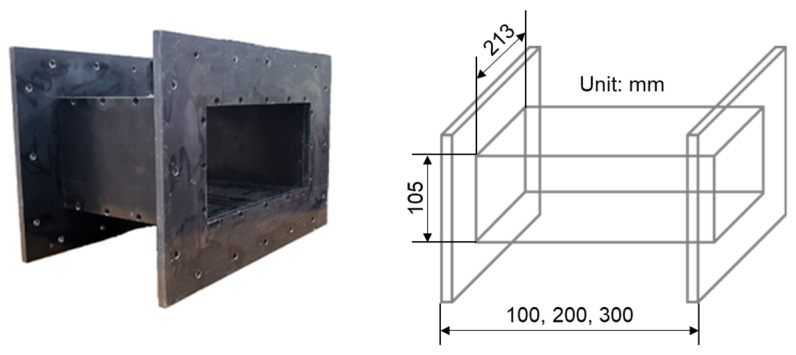
Specimen geometry and test setup for electrical measurements.

The specimens for compressive strength evaluation were fabricated in a cylindrical shape with a diameter of 100 mm and a height of 200 mm, and those for flexural strength evaluation were fabricated in the form of a rectangular parallelepiped with a width of 100 mm, height of 100 mm, and a length of 400 mm. The specimens for electrical conductivity evaluation had the same size as those for flexural strength evaluation. Silver-plated copper plates were used as electrodes for resistance measurements to reduce the influence of contact resistance and corrosion, and four electrodes were placed at 100 mm intervals. Figure 2 shows the specimen geometry for resistance measurements. The specimens used for the evaluation of the electromagnetic shielding effectiveness were fabricated using the mold shown in Figure 3. They had the shape of a rectangular parallelepiped with a width of 213 mm, height of 105 mm, and a thickness of 100, 200, or 300 mm.

### 2.4. Experimental Method

The compressive strength was measured using three specimens at each level in accordance with the American Society for Testing and Materials (ASTM) C39 [32] “Standard Test Method for Compressive Strength of Cylindrical Concrete Specimens.” The flexural strength was evaluated using three specimens in accordance with ASTM C 1609 [33] “Standard Test Method for Flexural Performance of Fiber-Reinforced Concrete (Using Beam with Third-Point Loading),” and it was calculated using Equation (2).

(2) $$f=\frac{PL}{bd^{2}}$$

The electrical resistance was measured using a digital multimeter 34465A (Keysight, Santa Rosa, CA, USA) and a two-probe method. Three measurements were performed for two electrodes from the outer electrodes. In addition, measurements were acquired after 28 and 208 days to examine the influence of the moisture content, and the measured direct current (DC) resistance was used to calculate the electrical conductivity, as shown in Equation (3) [3,34].

(3) $$\sigma =\frac{1}{\rho }=\frac{1}{R}\times \frac{L}{S}$$

where σ is the electrical conductivity (S/cm), ρ is the electrical resistivity (Ω∙cm), R is the electrical resistance (Ω), S is the cross-sectional area of the specimen in contact with the electrode (cm^2^), and L is the distance between electrodes (cm). 

**Figure 4:**
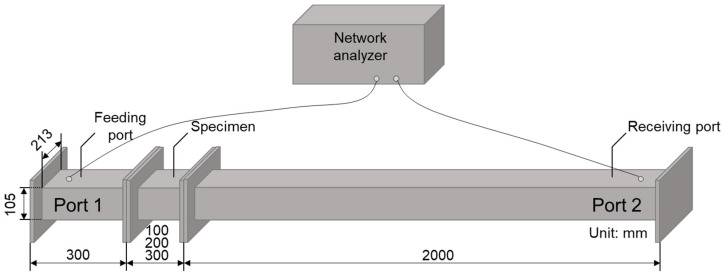
Test setup for electromagnetic shielding effectiveness measurements.

Figure 4 shows the test setup for measurements of the electromagnetic shielding effectiveness. The waveguides used for the electromagnetic shielding effectiveness were designed from aluminum (thickness: 8 mm), and those could be separated by 50 mm from the ground based on considerations of the coupling of the specimen mold during measurements. The internal size of the waveguide of port 1 had a width of 213 mm, height of 105 mm, and a length of 400 mm. The internal size of the waveguide of port 2 was the same as that of port 1, with the exception of its length which was equal to 2000 mm. The E8356A network analyzer (Agilent, Santa Clara, CA, USA) was used for measurements. Ports 1 and 2 were connected to the terminals of the instrument, and the test was conducted in the frequency range of 0.85–1.0 GHz. The shielding effectiveness was calculated using Equation (4) [35,36].

(4) $$\mathrm{SE}\left(\mathrm{dB}\right)=10log\left(\frac{P_{I}}{P_{T}}\right)$$

where SE is the shielding effectiveness of the tested specimen, $P_{I}$ is the incident power, and $P_{T}$ is the transmitted power.

**Figure 5:**
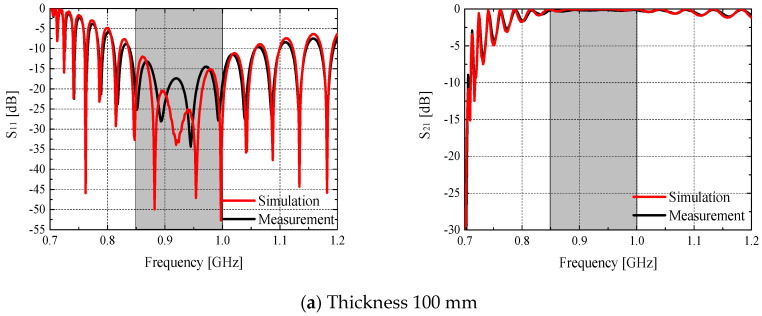

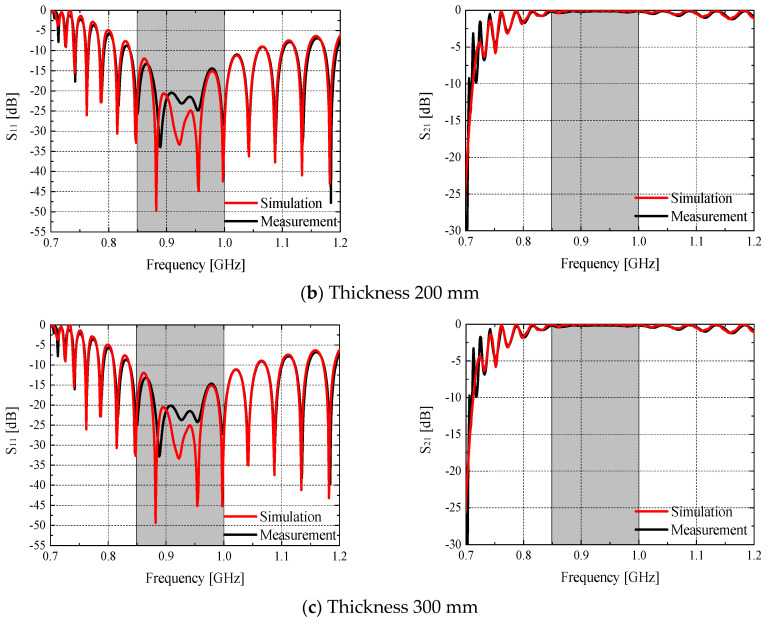
S-parameters of waveguides according to specimen mold thickness.

Table 6 and Figure 5 show the S-parameters of the waveguides as a function of the specimen mold thickness. It was measured in the absence of a shielding material inside the mold. S_11_ is the reflection coefficient and represents the ratio of the power returned to port 1 by unmatched sites (e.g., conductor walls and substances whose impedances do not match that of the antenna) to the incident power. S_21_ is the transmittance coefficient and represents the transfer characteristics of the incident voltage at port 1 to port 2; it is used to identify the electromagnetic shielding effectiveness. The transmittance coefficient S_21_ of the waveguides was close to 0 dB in the absence of shielding material, thus indicating that no signal escaped from the inner parts of the waveguides, and that the influence of external signals was minimized.

## 3. Experimental Results and Discussion

### 3.1. Compressive and Flexural Properties 

**Figure 6:**
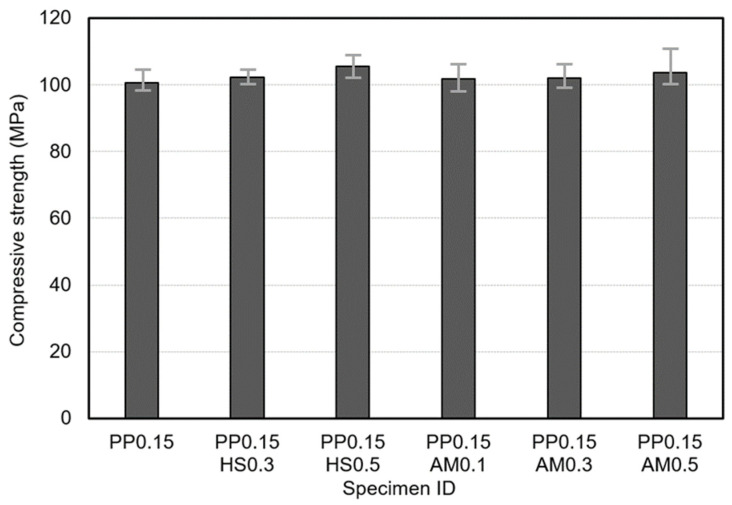
Compressive strength responses of studied samples.

**Figure 7:**
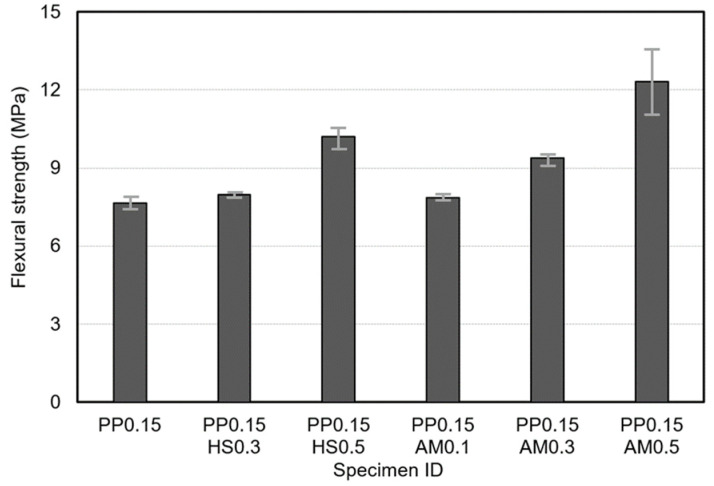
Flexural strength responses of studied samples.

Figure 6 and Figure 7 show the compressive and flexural strength responses. There were no significant differences in compressive strength as a function of the type or content of steel fibers, but the specimens reinforced with hooked-end steel fibers or amorphous metallic fibers exhibited higher compressive strength responses than those reinforced only with polypropylene fibers. This result was similar to the previous study [28]. For all specimens, the compressive strength satisfied the target compressive strength as it ranged from 100 to 105 MPa. In the case of the flexural strength, however, there were significant differences depending on the type and content of the steel fibers. In particular, the specimens reinforced with amorphous metallic fibers yielded higher flexural strengths than those reinforced with hooked-end steel fibers. The amorphous metallic fibers exhibited an excellent bridging effect owing to the increased adhesion efficiency with the matrix because they had a larger specific surface area than hooked-end steel fibers, and relatively more fibers were thus added at the same content. Previous studies also mentioned improvements in flexural and tensile strengths owing to the special geometry and physical properties of amorphous metallic fibers [22,24], and a similar tendency was also observed in this study. 

### 3.2. Electrical Conductivity 

**Figure 8:**
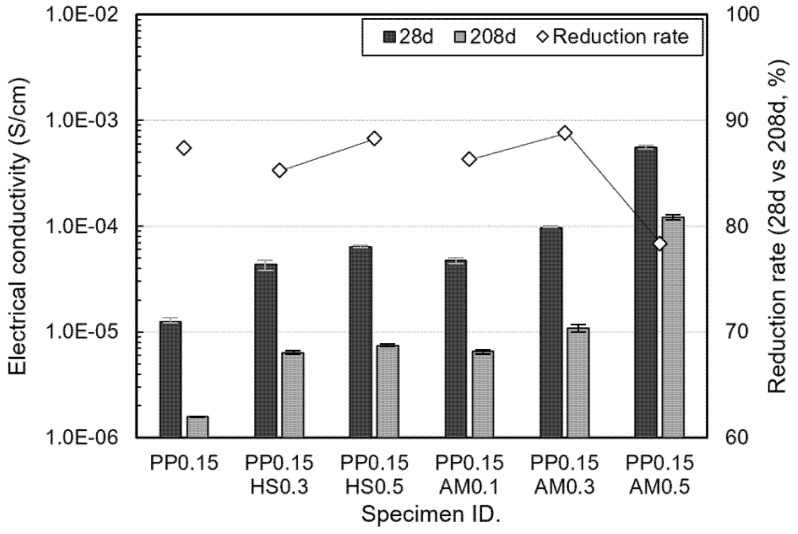
Conductivity of fibers of tested specimens.

Figure 8 shows the electrical conductivity of the specimens cured for 28 and 208 days and the conductivity reduction rate of the specimens cured for 208 days compared with those cured for 28 days. Regardless of the curing days, PP0.15 (with no steel fiber reinforcement) exhibited the lowest electrical conductivity. In the case of the specimens reinforced with hooked-end steel fibers and amorphous metallic fibers, the conductivity was significantly improved compared with PP0.15. This appears to be because the electrical conduction paths were formed by metal fibers. Although PP0.15AM0.1 had a lower content than PP0.15HS0.3, it exhibited an equivalent level of electrical conductivity. At the same content, the specimens reinforced with amorphous metallic fibers yielded higher electrical conductivities. Amorphous metallic fibers can be added in larger quantities at the same content because they have thin-plate shapes with large specific surface areas and low densities, even though their lengths are the same as those of the hooked-end steel fibers. Thus, it appears that amorphous metallic fibers achieve an excellent conductive network by forming many current movement paths as they are randomly placed inside concrete in large quantities. The specimens cured for 208 days exhibited a significant reduction in electrical conductivity compared with those cured for 28 days. This can be explained by the influence of the moisture content in the specimens. While the specimens cured for 28 days had moisture contents of around 5.9%, those cured for 208 days exhibited moisture contents of around 2.7%, which was less than half of the content of the specimens cured for 28 days. A previous study reported that the moisture inside the cement-based materials affects ionic conduction [37]. 

Therefore, it appears that the conductivity decreases as the conductive paths decrease owing to a reduction in the moisture content, regardless of the specimen type. While the specimens (other than PP0.15AM0.5) yielded conductivity reduction rates between 85 and 90%, PP0.15AM0.5 exhibited a lower conductivity reduction rate of around 78%. Previous studies reported that electrical resistance remains constant, regardless of the formation of moisture or cement hydration products, once a percolated conductive network was formed [3,38]. It appears that PP0.15AM0.5 was affected to a lesser extent by moisture as it formed a network close to a percolated conductive network, even though it had the same content as PP0.15HS0.5. 

### 3.3. Electromagnetic Shielding Effectiveness

**Figure 9:**
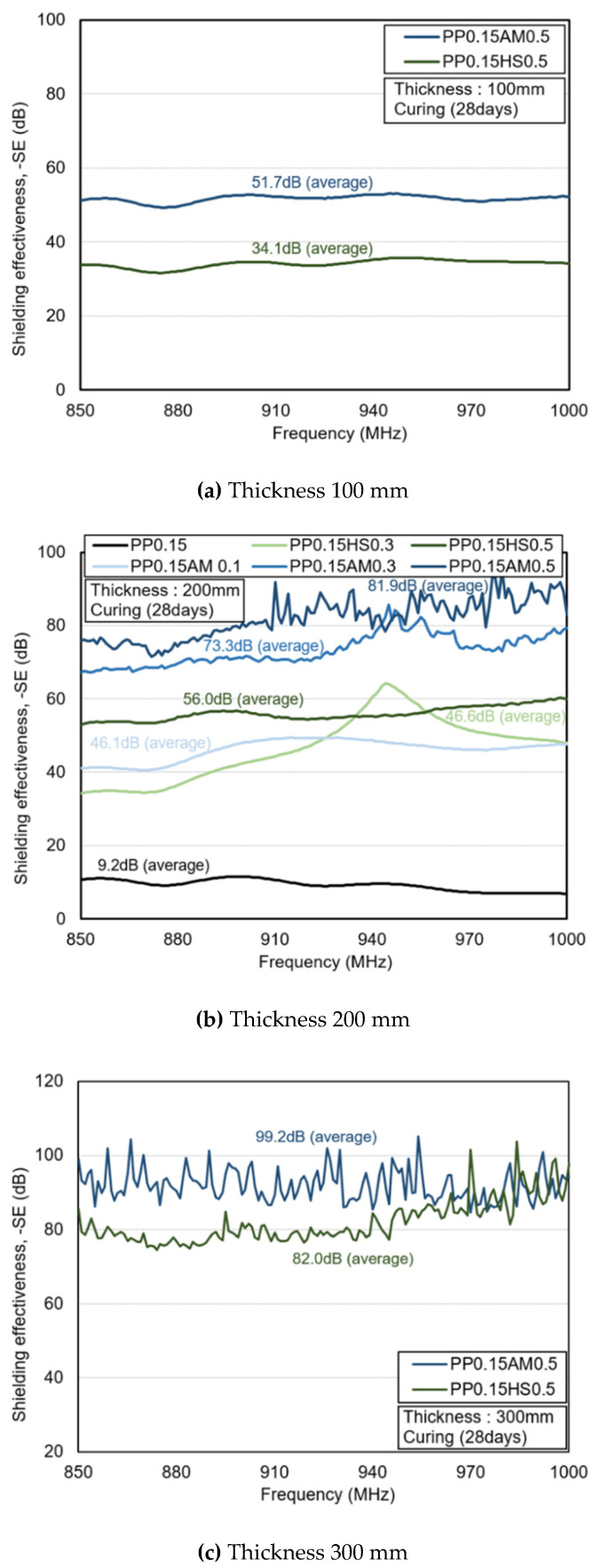
Shielding effectiveness of tested specimens (curing age: 28 days).

Figure 9 shows the electromagnetic shielding effectiveness of the specimens at 28 days of age. At a thickness of 100 mm, the electromagnetic shielding effectiveness of PP0.15AM0.5 was around 51.7 dB, which was around 52% higher than that of PP0.15HS0.5 (approximately 34.1 dB). The reason is that PP0.15AM0.5 considerably reduced the electromagnetic wave intensity by inducing a large current with an excellent conductive network compared to PP0.15HS0.5. 

At a thickness of 200 mm, the overall shielding effectiveness was significantly improved compared to that at 100 mm as the electromagnetic wave reflection and absorption losses were considerably increased owing to the increased thickness. The shielding effectiveness of PP0.15AM0.5 was around 46% higher than that of PP0.15HS0.5. PP0.15AM0.3 exhibited high shielding effectiveness of around 31%, even though it had a lower content than PP0.15HS0.5. PP0.15AM0.1 and PP0.15HSF0.3 yielded similar shielding effectiveness values equal to 46.1 and 46.6 dB, respectively. Meanwhile, PP0.15 yielded a shielding effectiveness of around 9.2 dB, thus confirming that it is difficult to achieve the expected shielding effectiveness if conductive fibers are not added. 

At a thickness of 300 mm, PP0.15AM0.5 exhibited around 21% higher shielding effectiveness than PP0.15HS0.5. As the thickness increased, the difference in the shielding effectiveness demonstrated a tendency to decrease as a function of the electrical conductivity difference. Since the specimen used in this study is a composite in which various materials are mixed, resonance may occur at a specific frequency depending on the material properties and thickness of the specimen, and reflection may occur at a specific frequency. In particular, when the dB value of PP0.15AM0.5 is viewed as a linear value, it is a very small value of about −80dB, which is close to noise. Therefore, fluctuations seem to occur owing to the increase in sensitivity.

**Figure 10:**
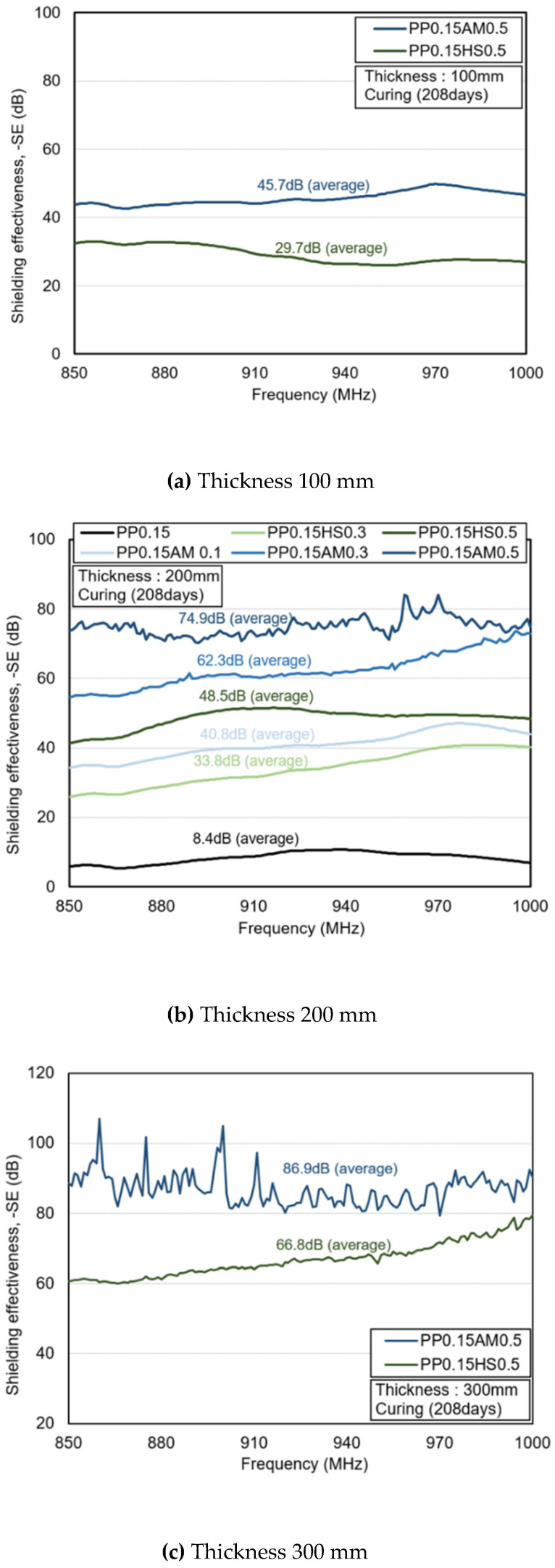
Shielding effectiveness (curing age: 208 days).

Figure 10 shows the electromagnetic shielding effectiveness of the specimens after 208 days. Overall, the shielding effectiveness decreased compared to that of the specimens after 28 days. This appears to be because the electrical conductivity decreased owing to a reduction in the moisture content inside the specimens. At a thickness of 100 mm, PP0.15AM0.5 exhibited a higher electromagnetic shielding rate than PP0.15HS0.5 (by around 54%), confirming a larger difference compared with the specimens at 28 days of age. At a thickness of 200 mm, PP0.15AM0.5 yielded a higher shielding rate than PP0.15HS0.5 by around 54%, and resulted in a larger difference compared with those at 28 days of age. Meanwhile, PP0.15AM0.1 and PP0.15HS0.3, which exhibited similar shielding effectiveness values at 28 days, had a clear difference in shielding effectiveness at 208 days, thus confirming an excellent shielding efficiency outcome attributable to the amorphous, metallic fiber reinforcement. 

At a thickness of 300 mm, PP0.15AM0.5 exhibited around 30% higher shielding effectiveness than PP0.15HS0.3, thus confirming that concrete reinforced with amorphous metallic fibers had a low-shielding effectiveness reduction rate after 208 days, regardless of the thickness. Both hooked-end steel fibers and amorphous metallic fibers improved the shielding effectiveness as their content increased. In particular, it was confirmed that amorphous metallic fibers can obtain higher electromagnetic shielding effectiveness at a lower content compared with hooked-end steel fibers. Although the test was conducted using waveguides, PP0.15AM0.5 yielded results that exceeded 80 dB at a thickness of 300 mm (850 to 1000 MHz); these results conform to the shielding effectiveness criterion suggested by MIL-STD-188-125-1 [39], which confirmed the applicability of amorphous metallic fibers as electromagnetic shielding materials. 

**Figure 11:**
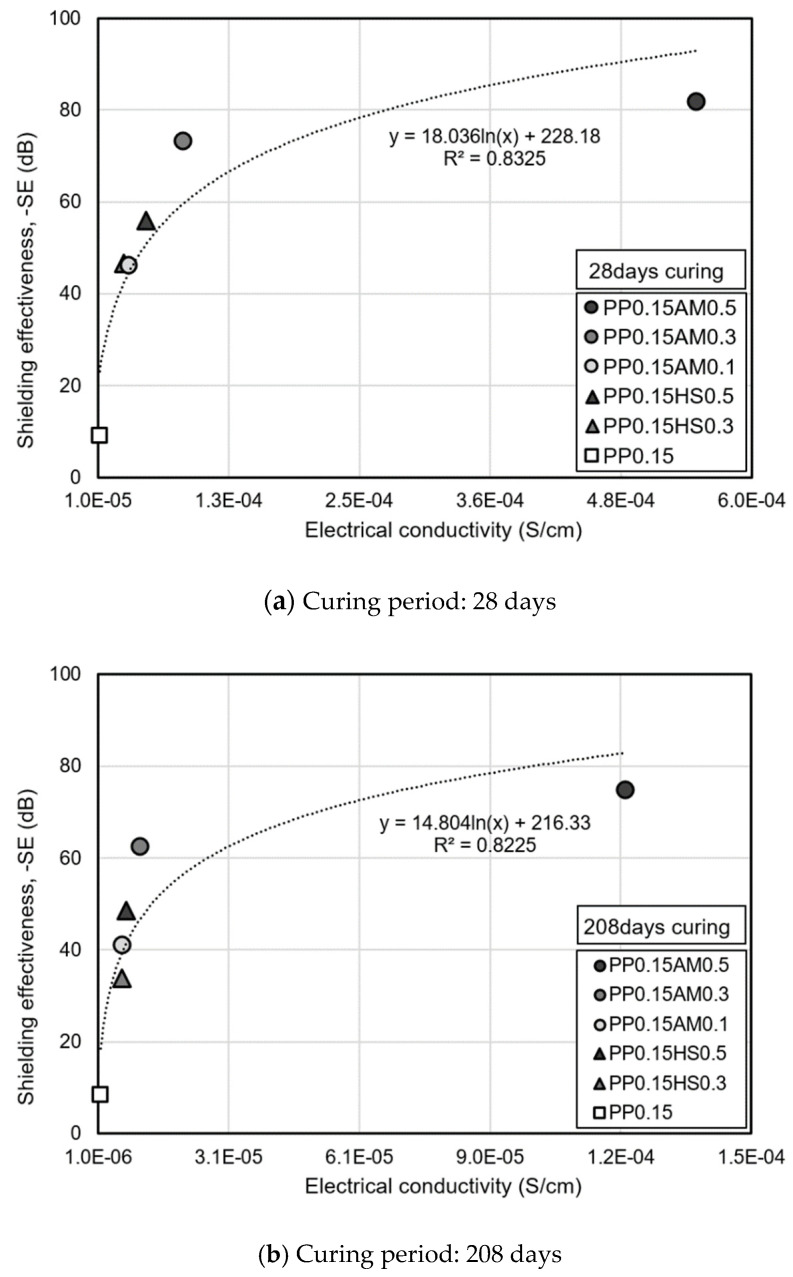
Shielding effectiveness as a function of electrical conductivity.

Figure 11 shows the shielding effectiveness according to the electrical conductivity based on the electromagnetic shielding effectiveness at a thickness of 200 mm. After 28 days, PP0.15AM0.1 yielded around 3.2 times higher conductivity and 5 times higher shielding effectiveness than PP0.15. In the case of PP0.15AM0.5, the conductivity was approximately 46 times higher, but the shielding effectiveness was only 8.9 times higher compared to PP0.15. This indicates that when conductive fibers were added into concrete with very low conductivity, the shielding effectiveness increased abruptly at a low content owing to the formation of a conductive network, but the shielding effectiveness leads to the saturation level when a percolation conductive network is approached owing to the increased content. A previous study also reported that the addition of conductive fibers into cementitious materials results in an abrupt improvement in conductivity and electromagnetic shielding performance, but the improvement effect according to the increased content decreased beyond a certain content [40]. In addition, it was reported that an increase in the length or aspect ratio of the conductive fibers is favorable for forming a conductive network and improves the electromagnetic shielding effectiveness [19,41,42]. Meanwhile, the amorphous metallic fibers and hooked-end steel fibers used in this study have the same length and length/equivalent diameter ratio value, but the concrete reinforced with the amorphous metallic fibers clearly exhibited higher electrical conductivity and electromagnetic shielding performance. This may be attributed to the much larger specific surface area and quantity of amorphous metallic fibers compared to those of hooked-end steel fibers.

After 208 days, the electrical conductivity–shielding effectiveness relationship also showed a similar tendency. Compared to PP0.15AM0.5, the other specimens exhibited significantly reduced electrical conductivity and shielding performance owing to the reduction in moisture content caused by long-term aging. As described above, when 0.5 vol.% of amorphous metallic fibers was added, it appears that the electromagnetic shielding effectiveness was affected to a lesser extent because a percolated conductive network was formed, and the influence of moisture or hydration products decreased.

Consequently, amorphous metallic fibers form an excellent conductive network at a lower content compared to that formed by hooked-end steel fibers because the former have a larger specific surface area and quantity at the same content. This allows amorphous metallic fibers to attain relatively higher shielding effectiveness than hooked-end steel fibers.

## 4. Conclusions

In this study, the electromagnetic shielding effectiveness of amorphous metallic fiber-reinforced, high-strength concrete was evaluated using waveguides. The inferred conclusions were as follows:

1. Regardless of the reinforcing fibers, the flexural strength of fiber-reinforced, high-strength concrete was improved as the content of fibers increased owing to an improvement in the bridging effect. In particular, amorphous metallic fibers significantly improved the flexural strength compared to hooked-end steel fibers owing to the improved adhesion efficiency with the matrix. This was attributed to their larger surface area as thin plates and the fact that relatively more fibers were added at the same content.
2. Amorphous metallic fibers are favorable for forming a conductive network because they can be added in large quantities owing to their low density, and have a large specific surface area as thin plates, even though they have the same length and length/equivalent diameter ratio value as hooked-end steel fibers. Therefore, it can be said that amorphous metallic fibers form a percolation conductive network at a lower content compared with hooked-end steel fibers.
3. The addition of metal fibers improved the electromagnetic shielding effectiveness (frequency range: 850–1000 Hz) owing to an improvement in electrical conductivity. Additionally, the shielding effectiveness of more than 80 dB (based on a thickness of 300 mm) was observed at a low moisture content after 208 days when amorphous metallic fibers (0.5 vol.%) were added. In addition, similar to the electrical conductivity, the efficient shielding effectiveness can be expected from amorphous metallic fibers at a low content compared with hooked-end steel fibers.
4. Regardless of the age, the electromagnetic shielding effectiveness clearly improved as the electrical conductivity increased, and it can be expected that the shielding effectiveness will approach the saturation level when the fiber volume fraction of amorphous metallic fibers exceeds 0.5 vol.%. Meanwhile, given that the electrical conductivity and electromagnetic shielding effectiveness decrease owing to a reduction in the moisture content of the specimen, it is necessary to reduce the amount of moisture to conservatively evaluate the electromagnetic shielding performance.

In future research, the effects of the addition of amorphous metallic fibers will be analyzed in detail by identifying the percolation threshold of the electrical conductivity based on the preparation of amorphous metallic fiber-reinforced cement composites with various lengths and high contents, and by evaluating the electromagnetic shielding performance using a shielding room. In addition, it is necessary to analyze the electrical conductivity and electromagnetic shielding performance of steel fiber-reinforced cement composites by constructing a database based on a large number of tests.

## Figures and Tables

**Table 1:** Physical properties of the used fibers.

Mechanical Properties
Length: 15 mm, diameter: 20 µm, density: 0.91 g/cm^3^, melting point: 170 °C
Length: 30 mm, width: 1.6 mm, thickness: 29 µm, density: 7.2 g/cm^3^, tensile strength: 1400 MPa, specific surface area: 9.6 m^2^/kg
Length: 30 mm, diameter: 0.25 mm, density: 7.85 g/cm^3^, tensile strength: 1140 MPa, specific surface area: 1.0 m^2^/kg

**Table 2:** Physical properties of the materials used in this study.

Materials	Mechanical Properties
Cement	Ordinary Portland cement, density: 3150 kg/m^3^, fineness: 320 m^2^/kg
Silica fume	Density: 2500 kg/m^3^, fineness: 20,000 m^2^/kg
Ground granulated blast-furnace slag	Density: 2500 kg/m^3^, fineness: 600 m^2^/kg
Coarse aggregate	Crushed granitic aggregate, maximum size: 20 mm, density: 2700 kg/m^3^, absorption: 0.9%
Fine aggregate	River sand, density: 2650 kg/m^3^, absorption: 1%, fineness modulus: 2.6
Super plasticizer	Polycarboxylic acid type

**Table 3:** Experimental plan.

Identity ^1^	Fiber Type and Volume Fraction			Evaluation Items
	PP	HS	AM	
PP0.15	0.15	-	-	Compressive strength (MPa) Flexural strength (MPa) Electrical conductivity (S/cm) Electromagnetic shielding effectiveness (dB)
PP0.15HS0.3		0.3	-	
PP0.15HS0.5		0.5	-	
PP0.15AM0.1		-	0.1	
PP0.15AM0.3		-	0.3	
PP0.15AM0.5		-	0.5	

^1^ PP: polypropylene fiber, AM: amorphous metallic fiber, HS: Hooked-end steel fiber PP0.15AM0.5: polypropylene fiber (0.15 vol.%) and amorphous metallic fiber (0.5 vol.%)-reinforced high-strength concrete.

**Table 4:** Mix proportions of the fiber-reinforced high-strength concrete.

W/B	C/B	SF/B	GGBS/B	S/a	Fibers (kg)		
					Polypropylene Fiber	Amorphous Metallic Fiber	Hooked-End Steel Fiber
0.19	0.7	0.15	0.15	0.45	1.4 (0.15 vol.%)	7.2 (0.1 vol.%) 21.6 (0.3 vol.%) 36.0 (0.5 vol.%)	23.6 (0.3 vol.%) 39.3 (0.5 vol.%)

W: Water, B (C + SF + GGBS): Binder, C: Cement, SF: Silica fume, GGBS: Ground granulated blast-furnace slag, S: Fine aggregate, a (S + G (coarse aggregate)): Total amount of aggregate.

**Table 5:** Moisture content of specimens.

Curing Age (Days)	Moisture Content (%)					
	PP0.15	PP0.15 HS0.3	PP0.15 HS0.5	PP0.15 AM0.1	PP0.15 AM0.3	PP0.15 AM0.5
28	6.0	5.8	5.9	5.9	5.8	5.8
208	2.7	2.8	2.7	2.6	2.6	2.7

**Table 6:** S-parameters of waveguides as a function of specimen mold thickness.

Frequency (GHz)		0.85		0.925		1.0	
		Simulation	Measurement	Simulation	Measurement	Simulation	Measurement
Thickness: 100 mm	S_11_ (dB)	−20.73	−23.44	−32.01	−17.81	−28.74	−18.68
	S_21_ (dB)	−0.05	−0.14	−0.03	−0.17	−0.02	−0.16
Thickness: 200 mm	S_11_ (dB)	−20.66	−25.52	−32.21	−23.06	−29.52	−25.69
	S_21_ (dB)	−0.1	−0.16	−0.06	−0.15	−0.05	−0.13
Thickness: 300 mm	S_11_ (dB)	−20.6	−24.9	−32.24	−23.61	−28.86	−24.81
	S_21_ (dB)	−0.1	−0.17	−0.06	−0.16	−0.05	−0.15
